# Supplementary material for: Localization of Viral Epitope-Specific CD8 T Cells during Cytomegalovirus Latency in the Lungs and Recruitment to Lung Parenchyma by Airway Challenge Infection
Source: Life (Basel). 2021 Sep 4;11(9):918. doi: 10.3390/life11090918 (PMC8467276; doi:10.3390/life11090918)
Supplement: Supplementary file 1 [file life-11-00918-s001.zip › life-1360124-supplementary.pdf]

life

Supplementary Materials

Localization of Viral Epitope-Specific CD8 T Cells During Cytomegalovirus Latency in the Lungs and Recruitment to Lung Parenchyma by Airway Challenge Infection

Franziska Blaum [1], Dominika Lukas [2], Matthias J. Reddehase [1,*] and Niels A. W. Lemmermann [1]

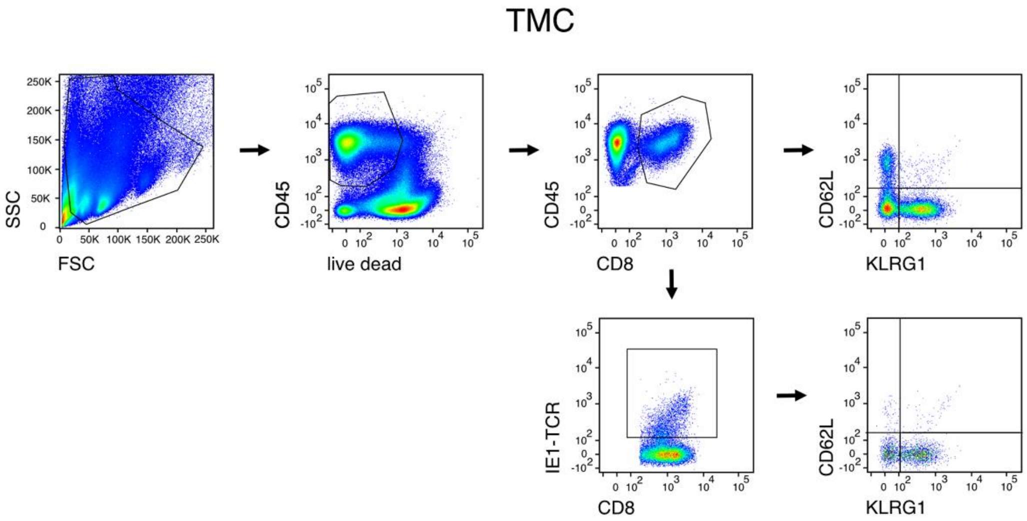

Figure S1. Representative example of the complete gating strategy for cells in the TMC to exclude contaminating CD45- lung tissue cells from the analysis.
